# Supplementary material for: Plasticity and evolutionary convergence in the locomotor skeleton of Greater Antillean Anolis lizards
Source: eLife. 2020 Aug 13;9:e57468. doi: 10.7554/eLife.57468 (PMC7508556; doi:10.7554/eLife.57468)
Supplement: Supplementary file 1. [file elife-57468-supp1.docx]

Plasticity and evolutionary convergence in the locomotor skeleton of Greater Antillean Anolis lizards

Nathalie Feiner^a,1^, Illiam S.C. Jackson^a,2^, Kirke L. Munch^b^, Reinder Radersma^a,3^ & Tobias Uller^a^

^a^Department of Biology, Lund University, 223 62 Lund, Sweden

^b^School of Biological Sciences, University of Tasmania, Hobart TAS 7001, Australia

^1^To whom correspondence may be addressed. Email: [nathalie.feiner@biol.lu.se](mailto:nathalie.feiner@biol.lu.se)

^2^Present address: College of Natural Sciences, University of Texas in Austin, US

^3^Present address: Biometris, Wageningen University & Research, Wageningen, The Netherlands.

**This supplementary file 1 includes:**

Supplementary Files 1A-1L (supplementary tables)

**Supplementary tables**

**Supplementary file 1A.** Anatomical position of landmarks used to quantify the shape of pectoral and pelvic girdles. Landmarks for capturing variation in the pectoral girdles were partially adopted from Tinius and Russell (2014) and for the pelvic girdle from Tinius et al., (2018).

| Girdle | Landmark | Anatomical position |
| --- | --- | --- |
| Pectoral | 1 | Lateral apex of the primary curvature of the clavicle |
|  | 2 | Anterior extremity of the clavicle |
|  | 3 | Anterior-most point of the dorsal edge of the interclavicle |
|  | 4 | Lateroposterior extremity of the epicoracoid |
|  | 5 | Lateral extremity of the interclavicle |
|  | 6 | Dorsoposterior-most point of contact between the clavicle and the scapulocoracoid |
|  | 7 | Anterior-most point of contact between the suprascapula and the scapula |
|  | 8 | Posterior-most point of contact between the suprascapula and the scapula |
|  | 9 | Ventroanterior extremity of the scapular ray |
|  | 10 | Posterior extremity of the primary coracoid fenestra |
|  | 11 | Posterior extremity of the scapulacoracoid fenestra |
|  | 12 | Anteromedial extremity of the presternum |
|  | 13 | Articulatory point between the intersternum and the first sternal rib |
|  | 14 | Articulatory point between the intersternum and the second sternal rib |
|  | 15 | Articulatory point between the intersternum and the third sternal rib |
|  | 16 | Lateral-most point of the dorsal edge of the coracosternal groove |
|  | 17 | Dorsal extremity of the dorsolateral process of the presternum |
|  | 18 | Dorsal extremity of the superior glenoid buttress |
| Pelvic | 1 | Anterior extremity of the epipubis |
|  | 2 | Posterior extremity of the epipubis |
|  | 3 | Ventrolateral extremity of the pubic tubercle |
|  | 4 | Ventroposterior extremity of the obturator foramen |
|  | 5 | Anterior extremity of the proischiadic cartilage |
|  | 6 | Anterolateral-most point of contact between the proischiadic cartilage and the ischium |
|  | 7 | Posterior extremity of the thyroid fenestra |
|  | 8 | Lateral extremity of the thyroid fenestra |
|  | 9 | Posterolateral-most point of contact between the hypoischium and the ischium |
|  | 10 | Posterior extremity of the ischiadic tuberosity |
|  | 11 | Anterior-most point of the curvature between the ischiadic tuberosity and the acetabulum |
|  | 12 | Ventroposterior extremity of the acetabulum |
|  | 13 | Anterodorsal extremity of the preacetabular process |
|  | 14 | Dorsal-most point of contact between the ilium and the epiphyseal tuberosity |
|  | 15 | Ventral-most point of contact between the ilium and the epiphyseal tuberosity |
|  | 16 | Anterior extremity of the acetabulum |
|  | 17 | Apex of the anterodorsal curve of the anterior portion of the ilium |
|  | 18 | Anterior-most point of the curvature between the acetabulum and epiphyseal tuberosity |

**Supplementary file 1B.** Estimations of the strength of convergence for each ecomorph class using the Wheatsheaf index as a metric. The table reports the calculated Wheatsheaf index per ecomorph and dataset, together with the lower and upper boundaries (95% confidence interval) and associated *P*-values resulting from 10 000 simulations. Significant *P*-values are highlighted bold.

| Dataset | Ecomorph | w index | Lower boundary | Upper boundary | *P*-value |
| --- | --- | --- | --- | --- | --- |
| All traits | trunk-ground | 1.097 | 1.086 | 1.117 | 0.138 |
| (N = 132) | trunk-crown | 1.030 | 1.018 | 1.053 | 0.640 |
|  | crown-giant | 1.204 | 1.192 | 1.270 | 0.135 |
|  | twig | 1.063 | 1.050 | 1.152 | 0.731 |
|  | grass-bush | 1.056 | 1.044 | 1.078 | 0.517 |
|  | trunk | 0.961 | 0.948 | 0.999 | 0.927 |
| Pectoral shape | trunk-ground | 1.164 | 1.148 | 1.182 | 0.062 |
| (N = 54) | trunk-crown | 1.052 | 1.039 | 1.080 | 0.546 |
|  | crown-giant | 1.206 | 1.165 | 1.299 | 0.204 |
|  | twig | 1.135 | 1.117 | 1.235 | 0.549 |
|  | grass-bush | 1.030 | 1.020 | 1.052 | 0.658 |
|  | trunk | 0.838 | 0.806 | 0.876 | 0.950 |
| Pelvic shape | trunk-ground | 1.049 | 1.037 | 1.069 | 0.349 |
| (N = 54) | trunk-crown | 0.989 | 0.977 | 1.007 | 0.869 |
|  | crown-giant | 1.207 | 1.194 | 1.273 | 0.135 |
|  | twig | 0.981 | 0.970 | 1.049 | 0.908 |
|  | grass-bush | 1.109 | 1.096 | 1.127 | 0.201 |
|  | trunk | 1.112 | 1.100 | 1.169 | 0.536 |
| Limb length | trunk-ground | 1.027 | 1.013 | 1.043 | 0.496 |
| (N = 15) | trunk-crown | 1.097 | 1.080 | 1.133 | 0.280 |
|  | **crown-giant** | **1.486** | **1.467** | **1.559** | **0.014** |
|  | twig | 0.969 | 0.955 | 1.028 | 0.884 |
|  | grass-bush | 1.017 | 1.002 | 1.049 | 0.713 |
|  | trunk | 1.025 | 1.012 | 1.073 | 0.759 |
| Bone thickness | trunk-ground | 1.103 | 1.075 | 1.119 | 0.293 |
| (N = 8) | trunk-crown | 1.054 | 1.032 | 1.087 | 0.510 |
|  | crown-giant | 0.913 | 0.890 | 0.959 | 0.786 |
|  | twig | 1.416 | 1.378 | 1.511 | 0.185 |
|  | grass-bush | 0.936 | 0.919 | 0.959 | 0.844 |
|  | trunk | 1.339 | 1.299 | 1.415 | 0.244 |

**Supplementary file 1C.** ‘Signature traits’ of each ecomorph class and their corresponding *P-*values as estimated in the R package windex. Abbreviations: cort, cortical thickness; LM, landmark; pec, pectoral girdle; pel, pelvic girdle; pha1_f_length, first phalange of the fore-limb; pha2_f_length, second phalange of the fore-limb; pha1_h_length, first phalange of the hind-limb; pha2_h_length, second phalange of the hind-limb.

| trunk-ground | trunk-crown | crown-giant | twig | grass-bush | trunk |
| --- | --- | --- | --- | --- | --- |
| pec_LM6.Z  *P* = 0.005 | pec_LM2.Y  *P* = 0.036 | pec_LM2.X  *P* = 0.023 | pec_LM3.Y  *P* = 0.010 | pel_LM1.Z  *P* = 0.030 | pel_LM9.X  *P* = 0.032 |
| pec_LM13.Y  *P* = 0.016 | pec_LM11.X  *P* = 0.029 | pec_LM5.Y  *P* = 0.038 | pec_LM4.X  *P* = 0.023 | pel_LM10.X  *P* = 0.014 | pel_LM10.X  *P* = 0.022 |
| pec_LM14.Y  *P* = 0.013 | pec_LM12.Z  *P* = 0.007 | pec_LM5.Z  *P* = 0.041 | pec_LM16.X  *P* = 0.046 | pel_LM11.X  *P* = 0.014 | tibia_diameter  *P* = 0.030 |
| pec_LM15.X  *P* = 0.041 | femur_length  *P* = 0.037 | pec_LM7.Z  *P* = 0.027 | pec_LM17.X  *P* = 0.018 | pel_LM14.Y  *P* = 0.014 |  |
| pec_LM15.Z  *P* = 0.032 |  | pec_LM8.Y  *P* = 0.001 | pel_LM10.Z  *P* = 0.012 |  |  |
| pec_LM17.Y  *P* = 0.016 |  | pec_LM11.Z  *P* = 0.027 | pel_LM15.Y  *P* = 0.027 |  |  |
| pec_LM17.Z  *P* = 0.013 |  | pec_LM18.X  *P* = 0.012 | humerus_diameter  *P* = 0.028 |  |  |
| pel_LM11.Y  *P* = 0.004 |  | pel_LM2.Z  *P* = 0.022 | ulna_cort  *P* = 0.021 |  |  |
| pel_LM13.X  *P* = 0.047 |  | pel_LM12.X  *P* = 0.021 |  |  |  |
| pel_LM13.Z  *P* = 0.016 |  | pel_LM12.Z  *P* = 0.001 |  |  |  |
| pel_LM17.Z  *P* = 0.020 |  | pha1_f_length  *P* = 0.009 |  |  |  |
|  |  | pha2_f_length  *P* = 0.022 |  |  |  |
|  |  | pha1_h_length  *P* = 0.007 |  |  |  |
|  |  | pha2_h_length  *P* = 0.039 |  |  |  |
|  |  | tibia_length  *P* = 0.003 |  |  |  |

**Supplementary file 1D.** Pairwise, observed angles Θ and associated *P*-values. Shown are the degrees of the observed angles Θ and, in brackets, the *P*-values for significant parallelism and the *P*-value for significant orthogonal direction, respectively. Thus, observed angles Θ with the *P*-value below 0.025 (i.e. two-tailed test) mean that the divergence of ecomorphs pairs is indistinguishable from parallel on a given set of islands. Similarly, observed angles Θ with the *P*-value for orthogonal direction below 0.025 mean that the angle is significant different from orthogonal (i.e. not random). Note that only 53 out of the 90 ecomorph/island comparisons are possible due to three ecomorph/island categories that are missing (see Fig. 1A). Abbreviations: cg, crown-giant; gb, grass-bush; tc, trunk-crown; tg, trunk-ground; tr, trunk; tw, twig; Cub, Cuba; His, Hispaniola; Jam, Jamaica; PuR, Puerto Rico.

|  | His-Cub | His-Jam | His-PuR | Cub-Jam | Cub-PuR | Jam-PuR |
| --- | --- | --- | --- | --- | --- | --- |
| tr-gb | 37.12°  (0.026; <0.001) | NA | NA | NA | NA | NA |
| tr-tw | 75.56°  (<0.001; 0.005) | NA | NA | NA | NA | NA |
| tr-tc | 61.81°  (<0.001; <0.001) | NA | NA | NA | NA | NA |
| tr-cg | 53.30°  (<0.001; <0.001) | NA | NA | NA | NA | NA |
| tr-tg | 68.58°  (<0.001; <0.001) | NA | NA | NA | NA | NA |
| gb-tw | 57.47°  (<0.001; <0.001) | NA | 42.64°  (0.002; <0.001) | NA | 53.94°  (0.001; <0.001) | NA |
| gb-tc | 34.50°  (0.022; <0.001) | NA | 62.32°  (<0.001; <0.001) | NA | 61.11°  (<0.001; <0.001) | NA |
| gb-cg | 28.07°  (**0.037**; <0.001) | NA | 34.35°  (<0.001; <0.001) | NA | 35.31°  (0.001; <0.001) | NA |
| gb-tg | 43.83°  (<0.001; <0.001) | NA | 42.55°  (<0.001; <0.001) | NA | 41.31°  (0.002; <0.001) | NA |
| tw-tc | 62.68°  (<0.001; <0.001) | 41.20°  (0.007; <0.001) | 50.23°  (0.001; <0.001) | 78.18°  (<0.001; 0.024) | 72.62°  (<0.001; 0.005) | 42.28°  (**0.070**; <0.001**)** |
| tw-cg | 53.57°  (<0.001; <0.001) | 52.28°  (**0.048**; <0.001) | 46.40°  (<0.001; <0.001) | 78.93°  (<0.001; **0.058**) | 56.85°  (<0.001; <0.001) | 46.36°  (**0.158**; <0.001**)** |
| tw-tg | 50.69°  (<0.001; <0.001) | 52.98°  (<0.001; <0.001) | 48.64°  (<0.001; <0.001) | 59.55°  (<0.001; <0.001) | 57.39°  (<0.001; <0.001) | 53.37°  (<0.001; <0.001) |
| tc-cg | 34.49°  (<0.001; <0.001) | 70.12°  (<0.001; <0.001) | 44.86°  (<0.001; <0.001) | 72.45°  (<0.001; 0.001) | 56.14°  (<0.001; <0.001) | 62.35°  (0.001; <0.001) |
| tc-tg | 50.60°  (<0.001; <0.001) | 58.93°  (<0.001; <0.001) | 62.30°  (<0.001; <0.001) | 64.70°  (<0.001; 0.001) | 76.58°  (<0.001; 0.003) | 56.37°  (0.034; <0.001) |
| cg-tg | 48.22°  (<0.001; <0.001) | 66.69°  (0.007; <0.001) | 45.49°  (<0.001; <0.001) | 63.65°  (0.004; <0.001) | 40.63°  (0.001; <0.001) | 67.85°  (0.001; <0.001) |

**Supplementary file 1E.** Pairwise, observed ∆L and associated *P*-values. ∆L values with an associated *P*-value below 0.025 (significance level of 0.05 adjusted to a two-tailed test) mean that the ecomorph pairs have significantly different vector lengths. Abbreviations: cg, crown-giant; gb, grass-bush; tc, trunk-crown; tg, trunk-ground; tr, trunk; tw, twig; Cub, Cuba; His, Hispaniola; Jam, Jamaica; PuR, Puerto Rico.

|  | His-Cub | His-Jam | His-PuR | Cub-Jam | Cub-PuR | Jam-PuR |
| --- | --- | --- | --- | --- | --- | --- |
| tr-gb | 20.75 (<0.001) | NA | NA | NA | NA | NA |
| tr-tw | 43.72 (<0.001) | NA | NA | NA | NA | NA |
| tr-tc | 23.90 (<0.001) | NA | NA | NA | NA | NA |
| tr-cg | 23.49 (0.001) | NA | NA | NA | NA | NA |
| tr-tg | 15.73 (<0.001) | NA | NA | NA | NA | NA |
| gb-tw | 22.82 (<0.001) | NA | -7.30 (**1.000**) | NA | -30.13 (1.000) | NA |
| gb-tc | 4.82 (**0.053**) | NA | 1.22 (0.978) | NA | -3.60 (1.000) | NA |
| gb-cg | 5.76 (**0.039**) | NA | -11.47 (0.991) | NA | -17.23 (1.000) | NA |
| gb-tg | 14.34 (<0.001) | NA | 15.99 (0.002) | NA | 1.65 (0.891) | NA |
| tw-tc | 29.62 (<0.001) | 19.08 (**0.169**) | 8.29 (0.826) | -10.54 (1.000) | -21.33 (1.000) | -10.79 (0.995) |
| tw-cg | 6.08 (**0.073**) | 3.40 (**0.775**) | -20.51 (1.000) | -2.69 (0.921) | -26.59 (1.000) | -23.90 (0.997) |
| tw-tg | 14.68 (<0.001) | 11.54 (**0.361**) | -10.68 (0.999) | -3.14 (0.998) | -25.35 (1.000) | -22.22 (0.999) |
| tc-cg | 17.09 (<0.001) | 43.32 (<0.001) | 14.20 (0.262) | 26.23 (0.003) | -2.89 (0.942) | -29.12 (0.997) |
| tc-tg | 22.31 (<0.001) | 41.31 (<0.001) | 25.52 (<0.001) | 19.00 (0.013) | 3.21 (0.786) | -15.79 (0.997) |
| cg-tg | 1.82 (**0.188**) | 17.97 (0.006) | -9.22 (0.996) | 16.15 (0.099) | -11.04 (1.000) | -27.19 (0.998) |

**Supplementary file 1F.** Outlier traits explaining most of the difference between running/climbing specialists in environmentally induced (plasticity) and evolutionary divergence between ecomorphs. Outliers were identified using the function ‘scores’ in the R package ‘outliers’. Abbreviations: cort_thick, cortical thickness; LM, landmark; pec, pectoral girdle; pel, pelvic girdle; pha1_f_length, first phalange of the fore-limb; pha2_f_length, second phalange of the fore-limb; pha1_h_length, first phalange of the hind-limb; pha2_h_length, second phalange of the hind-limb; tg-tc, divergence between Cuban trunk-ground and trunk-crown ecomorphs.

| *A. carolinensis*  Plasticity | *A. sagrei*  Plasticity | tg-tc evolutionary divergence |
| --- | --- | --- |
| pec_LM2.X  t = 2.93 | pel_LM11.X  t = 2.57 | pha1_f_length  t = 11.41 |
| pel_LM17.X  *t =* 2.18 | pel_LM15.Y  *t =* 2.33 | pha2_f_length  *t =* 9.24 |
| humerus_length  *t =* 2.09 | pel_LM10.Z  *t =* 2.29 | pha1_h_length  *t =* 8.98 |
| pec_LM3.X  *t =* 2.06 | pel_LM1.Y  *t =* 2.06 | ulna_length  *t =* 8.91 |
| pec_LM13.X  *t =* 2.03 | pel_LM14.Y  *t =* 2.02 | tibia_length  *t =* 8.84 |
| pha2_f_length  *t =* 1.98 | tibia_length  *t =* -1.95 | pha4_f_length  *t =* 8.81 |
| pec_LM18.Z  *t =* -1.64 | pel_LM6.Y  *t =* -2.00 | pha2_h_length  *t =* 7.48 |
| pec_LM9.X  *t =* -1.72 | femur_length  *t =* -2.11 | femur_length  *t =* 7.14 |
| pel_LM17.Y  *t =* -1.75 | pel_LM13.Y  *t =* -2.42 | humerus_length  *t =* 6.58 |
| pec_LM18.X  *t =* -1.88 | pha5_h_length  *t* = -2.99 | pha3_h_length  *t =* 6.42 |
| pel_LM12.X  *t =* -1.89 |  | pel_LM3.Y  *t =* -4.87 |
| pec_LM5.Y  *t* = -2.16 |  | pec_LM8.Z  *t* = -4.94 |
| tibia_cort  *t* = -2.32 |  | pec_LM7.Z  *t* = -5.00 |
| pec_LM12.Y  *t* = -2.61 |  | pec_LM6.Y  *t =* -5.52 |
|  |  | pel_LM3.X  *t =* -5.84 |

**Supplementary file 1G.** Alignment between the runner/climber axes between environmentally induced and evolutionary divergent variation in morphology. The axis of evolutionary divergence describes the divergence between trunk-ground *versus* trunk-crown ecomorphs on the island of Cuba, which is the appropriate contrast since both *A. carolinensis* and *sagrei* originate from this island and belong to those ecomorphs. In brackets, the 95% confidence intervals from 999 bootstrappings of each data set are given. Note that all confidence intervals include or lie above 90° and are therefore not consistent with a significant alignment.

| Dataset | Plasticity *A. carolinensis* / Plasticity *A. sagrei* | Plasticity *A. carolinensis* / Evolutionary divergence | Plasticity *A. sagrei* / Evolutionary divergence |
| --- | --- | --- | --- |
| All traits (N = 132) | 80.84°  (69.65°-102.65°) | 81.22°  (70.97°-96.30°) | 105.96°  (88.67°-111.16°) |
| Most plastic traits in *A. sagrei* (N = 10) | NA | NA | 116.67°  (91.20°-130.41°) |
| Most plastic traits in *A. carolinensis* (N = 14) | NA | 87.84°  (65.57°-100.38°) | NA |
| Most evolutionary divergent traits (N = 15) | NA | 40.20°  (31.08°-100.00°) | 125.98°  (67.39°-138.93°) |

**Supplementary file 1H.** Output from linear mixed models examining the effect of principal components of morphology on perching behaviour and performance in *A. carolinensis*. Results are based on N = 45 male individuals.

|  | log(SVL) | PC1_Plasticity | PC2_Plasticity | PC1_EvolDiv | PC2_EvolDiv |
| --- | --- | --- | --- | --- | --- |
| Perching behavior |  |  |  |  |  |
| Average distance  from tip | *F*_(1,_ _38.63)_ = 0.31  *P* = 0.584 | *F*_(1,_ _38.51)_ = 1.36  *P* = 0.251 | ***F*_(1,_ _38.41)_ = 5.91**  ***P* = 0.020** | ***F*_(1,_ _38.94)_ = 9.63**  ***P* = 0.004** | *F*_(1,_ _38.75)_ = 0.87  *P* = 0.358 |
| Performance |  |  |  |  |  |
| Running track |  |  |  |  |  |
| log(TotalRaceTime) | *F*_(1,_ _39)_ = 0.33  *P* = 0.569 | *F*_(1,_ _39)_ = 1.66  *P* = 0.205 | *F*_(1,_ _39)_ = 0.38  *P* = 0.846 | *F*_(1,_ _39)_ < 0.00  *P* = 0.980 | *F*_(1,_ _39)_ = 0.34  *P* = 0.564 |
| log(VelocityMax) | *F*_(1,_ _36.48)_ = 1.76  *P* = 0.192 | *F*_(1,_ _38.95)_ = 0.80  *P* = 0.376 | *F*_(1,_ _38.95)_ = 0.21  *P* = 0.651 | *F*_(1,_ _38.59)_ = 0.65  *P* = 0.427 | *F*_(1,_ _37.08)_ = 0.48  *P* = 0.483 |
| Climbing track |  |  |  |  |  |
| log(TotalRaceTime) | *F*_(1,_ _37.90)_ = 0.02  *P* = 0.893 | *F*_(1,_ _34.66)_ = 0.24  *P* = 0.625 | *F*_(1,_ _33.42)_ = 0.36  *P* = 0.553 | *F*_(1,_ _36.97)_ = 0.44  *P* = 0.513 | *F*_(1,_ _37.88)_ = 1.87  *P* = 0.179 |
| log(VelocityMax) | *F*_(1,_ _38)_ = 0.18  *P* = 0.672 | *F*_(1,_ _38)_ < 0.00  *P* = 0.999 | *F*_(1,_ _38)_ = 2.41  *P* = 0.129 | *F*_(1,_ _38)_ < 0.00  *P* = 0.994 | *F*_(1,_ _38)_ = 1.05  *P* = 0.313 |

Significant effects are highlighted in bold. Cage ID a lizard was reared in was included as random effect.

**Supplementary file 1I.** Output from linear mixed models examining the effect of principal components of morphology on perching behaviour and performance in *A. sagrei*. Results are based on N = 71 male individuals.

|  | log(SVL) | PC1_Plasticity | PC2_Plasticity | PC1_EvolDiv | PC2_EvolDiv |
| --- | --- | --- | --- | --- | --- |
| Perching behavior |  |  |  |  |  |
| Average distance  from tip | *F*_(1,_ _53.82)_ = 2.91  *P* = 0.094 | *F*_(1,_ _59.02)_ = 0.82  *P* = 0.369 | *F*_(1,_ _59.96)_ = 0.47  *P* = 0.494 | *F*_(1,_ _56.01)_ = 0.24  *P* = 0.625 | *F*_(1,_ _57.85)_ = 0.61  *P* = 0.439 |
| Performance |  |  |  |  |  |
| Running track |  |  |  |  |  |
| log(TotalRaceTime) | *F*_(1,_ _54.95)_ = 2.04  *P* = 0.159 | *F*_(1,_ _59.55)_ = 1.08  *P* = 0.303 | *F*_(1,_ _61.01)_ = 0.04  *P* = 0.848 | *F*_(1,_ _56.21)_ = 0.07  *P* = 0.787 | ***F*_(1,_ _57.67)_ = 4.04**  ***P* = 0.049** |
| log(VelocityMax) | ***F*_(1,_ _47.57)_ = 4.61**  ***P* = 0.037** | *F*_(1,_ _54.66)_ = 0.11  *P* = 0.740 | *F*_(1,_ _56.87)_ = 1.32  *P* = 0.255 | *F*_(1,_ _49.66)_ = 0.19  *P* = 0.661 | *F*_(1,_ _51.47)_ = 3.51  *P* = 0.067 |
| Climbing track |  |  |  |  |  |
| log(TotalRaceTime) | ***F*_(1,_ _60.76)_ = 4.06**  ***P* = 0.048** | *F*_(1,_ _62.27)_ = 1.21  *P* = 0.275 | *F*_(1,_ _63.42)_ = 0.43  *P* = 0.516 | *F*_(1,_ _60.85)_ = 0.32  *P* = 0.571 | *F*_(1,_ _61.87)_ = 0.13  *P* = 0.724 |
| log(VelocityMax) | *F*_(1,_ _60.22)_ = 2.29  *P* = 0.135 | *F*_(1,_ _63.19)_ = 1.11  *P* = 0.295 | *F*_(1,_ _64.13)_ = 0.43  *P* = 0.515 | *F*_(1,_ _61.77)_ < 0.00  *P* = 0.979 | *F*_(1,_ _63.32)_ = 3.45  *P* = 0.068 |

Significant effects are highlighted in bold. Cage ID a lizard was reared in was included as random effect.

**Supplementary file 1J.** **Output from a Procrustes one-way ANOVA of the pectoral girdle.** Repeatability was assessed using the formula R = S^2^_A_/(S^2^_W_+S^2^_A_), where the among-individual variation S^2^_A_ is calculated by S^2^_A_ = (MS_Individual_ - MS_Error_)/2 and the within-individual variation S^2^_W_ is given by MS_Error_ (Arnqvist and Mårtensson 1998; Fruciano 2016). Repeatability was assessed on pectoral girdles of 10 randomly chosen individuals that were subjected to the entire landmarking procedure (including the thresholding of raw imaging data to extract mesh files) on two different occasions. Abbreviations: SS, sum of squares; MS, mean squares; df, degrees of freedom.

|  | SS | MS | df | R^2^ | *P*-value |
| --- | --- | --- | --- | --- | --- |
| Individual | 0.45 | 4.10 × 10^-2^ | 11 | 0.99 | <0.001 |
| Error | 0.01 | 5.17 × 10^-4^ | 12 | 0.01 | NA |

**Supplementary file 1K.** **Output from a Procrustes one-way ANOVA of the pelvic girdle.** Repeatability was assessed using the formula R = S^2^_A_/(S^2^_W_+S^2^_A_), where the among-individual variation S^2^_A_ is calculated by S^2^_A_ = (MS_Individual_ - MS_Error_)/2 and the within-individual variation S^2^_W_ is given by MS_Error_ (Arnqvist and Mårtensson 1998; Fruciano 2016). Repeatability was assessed on pelvic girdles of 10 randomly chosen individuals that were subjected to the entire landmarking procedure (including the thresholding of raw imaging data to extract mesh files) on two different occasions. Abbreviations: SS, sum of squares; MS, mean squares; df, degrees of freedom.

|  | SS | MS | df | R^2^ | *P*-value |
| --- | --- | --- | --- | --- | --- |
| Individual | 0.24 | 2.22 × 10^-2^ | 11 | 0.97 | <0.001 |
| Error | 0.01 | 6.55 × 10^-4^ | 12 | 0.03 | NA |

**Supplementary file 1L. Loadings of the principal components.** The ten most positive and most negative loading for each of the first three principal components (PCs) are given. Abbreviations: claw_f_length, claw of the forelimb; csize, centroid size of the pelvic girdle; cort, cortical thickness; LM, landmark; pec, pectoral girdle; pel, pelvic girdle; pha1_f_length, first phalange of the forelimb; pha1_h_length, first phalange of the hindlimb; pha2_h_length, second phalange of the hindlimb; pha3_h_length, third phalange of the hindlimb.

| PC1 | | PC2 | | PC3 | | PC4 | |
| --- | --- | --- | --- | --- | --- | --- | --- |
| trait | loading | trait | loading | trait | loading | trait | loading |
| pel_LM1.X | 0.182 | pec_LM16.Z | 0.149 | pel_LM2.Z | 0.186 | pec_LM4.Z | 0.214 |
| pel_LM1.Y | 0.180 | pel_LM13.Z | 0.147 | pel_LM1.Z | 0.163 | pec_LM17.Z | 0.198 |
| pel_LM2.Y | 0.159 | pel_LM4.Y | 0.145 | pel_LM16.X | 0.152 | pec_LM16.Z | 0.186 |
| humerus_length | 0.155 | pel_LM17.Z | 0.143 | pel_LM14.Y | 0.148 | pec_LM7.X | 0.169 |
| pel_LM2.X | 0.153 | pel_LM7.X | 0.143 | pec_LM3.X | 0.140 | pec_LM8.X | 0.158 |
| pel_LM6.Z | 0.143 | pec_LM18.Z | 0.136 | pel_LM11.X | 0.130 | pec_LM18.Z | 0.145 |
| pel_LM15.Y | 0.132 | pec_LM4.Z | 0.131 | pec_LM7.Y | 0.127 | pec_LM6.X | 0.144 |
| csize | 0.130 | pha1_f_length | 0.131 | pel_LM5.Z | 0.125 | pec_LM16.Y | 0.143 |
| claw_f_length | 0.128 | pec_LM5.X | 0.124 | pel_LM6.Z | 0.124 | pec_LM11.Z | 0.136 |
| pel_LM10.Y | 0.127 | pel_LM12.X | 0.121 | pec_LM8.Y | 0.123 | pec_LM10.Z | 0.119 |
| pec_LM7.Z | -0.112 | ulna_cort | -0.132 | pel_LM8.Z | -0.145 | pec_LM7.Z | -0.122 |
| pel_LM4.Z | -0.115 | pec_LM15.Z | -0.133 | pel_LM18.Z | -0.146 | pec_LM2.Z | -0.122 |
| pha3_h_length | -0.116 | pel_LM14.X | -0.143 | pec_LM12.Z | -0.151 | pel_LM18.X | -0.125 |
| pel_LM8.X | -0.131 | pel_LM10.X | -0.145 | pec_LM4.Y | -0.152 | pec_LM12.X | -0.133 |
| pel_LM3.Y | -0.140 | tibia_cort | -0.145 | pel_LM12.Y | -0.154 | pec_LM2.X | -0.144 |
| pel_LM16.Z | -0.144 | pec_LM14.X | -0.153 | pha3_h_length | -0.154 | pec_LM15.Z | -0.193 |
| pel_LM13.Y | -0.145 | pel_LM15.X | -0.158 | tibia_length | -0.156 | pec_LM14.Y | -0.203 |
| pel_LM6.X | -0.146 | humerus_cort | -0.171 | pel_LM11.Y | -0.157 | pec_LM14.Z | -0.203 |
| pel_LM3.X | -0.148 | femur_cort | -0.174 | pha1_h_length | -0.179 | pec_LM15.Y | -0.210 |
| pel_LM17.Y | -0.174 | pel_LM15.X | -0.183 | femur_length | -0.189 | pec_LM6.Z | -0.223 |

**References**

Arnqvist G, Mårtensson T. 1998. Measurement error in geometric morphometrics: Empirical strategies to assess and reduce its impact on measures of shape. *Acta Zoologica Academiae Scientiarum Hungaricae* **44**:73-96.

Fruciano C. 2016. Measurement error in geometric morphometrics. *Development Genes and Evolution* **226**:139-158.

Tinius A, Russell AP. 2014. Geometric morphometric analysis of the breast-shoulder apparatus of lizards: a test case using Jamaican anoles (Squamata: Dactyloidae). *Anat Rec (Hoboken)* **297**:410-432.

Tinius A, Russell AP, Jamniczky HA, Anderson JS. 2018. What is bred in the bone: Ecomorphological associations of pelvic girdle form in greater Antillean Anolis lizards. *Journal of Morphology* **279**:1016-1030.
